# HIV Prevention and Treatment Behavior Change and the Situated Information Motivation Behavioral Skills (sIMB) Model: A Qualitative Evaluation of a Community Health Worker Intervention in Rakai, Uganda

**DOI:** 10.1007/s10461-021-03391-w

**Published:** 2021-07-30

**Authors:** Rose Pollard, Caitlin E. Kennedy, Heidi E. Hutton, Jeremiah Mulamba, Ismail Mbabali, Aggrey Anok, Neema Nakyanjo, Larry W. Chang, K. Rivet Amico

**Affiliations:** 1 Division of Infectious Diseases, Department of Medicine, Johns Hopkins School of Medicine, Baltimore, MD, USA; 2 Department of International Health, Johns Hopkins Bloomberg School of Public Health, Baltimore, MD, USA; 3 Department of Psychiatry and Behavioral Sciences, Johns Hopkins School of Medicine, Baltimore, Maryland, USA; 4 Rakai Health Sciences Program, Rakai, Uganda; 5 Department of Health Behavior Health Education, University of Michigan, Ann Arbor, MI, USA

**Keywords:** HIV, community health worker, Uganda, behavioral intervention, situated-Information Motivation Behavioral Model

## Abstract

A community health worker (CHW) model can promote HIV prevention and treatment behaviors, especially in highly mobile populations. In a fishing community in Rakai, Uganda, the Rakai Health Sciences Program implemented a community health worker HIV intervention called Health Scouts. The situated Information, Motivation, and Behavioral Skills (sIMB) framework informed the design and a qualitative evaluation of the intervention. We interviewed 51 intervention clients and coded transcripts informed by sIMB framework dimensions. Clients reported that Health Scouts provided information about HIV prevention and treatment behaviors and helped them manage personal and social motivations to carry out health-promoting behavior. Prominent barriers which moved clients away from behavior change included daily pill burdens, anticipated stigma, serostatus disclosure, substance use at social gatherings, and anticipated reactions of partners. Our study adds to the evidence establishing CHWs as facilitators of behavior change, positioned to offer supportive encouragement and navigate contextualized circumstances.

## Introduction

Preventing and treating HIV in communities with high mobility in sub-Saharan Africa remains a challenge. Fishing communities have especially high mobility patterns as well as high HIV prevalence. These structural elements are associated with predisposing HIV risk factors among fishing communities in Uganda such as young age, substance use, and sexual risk behaviors (1, 2). In one large fishing landing site in Rakai, Uganda, HIV prevention messaging has been present since 1989, yet the community maintains an HIV prevalence rate of approximately 40%, six times higher than Uganda’s national prevalence rate (3–5). Given the availability of services and commodities for HIV prevention and treatment in fishing communities, a gap remains to promote further adoption of HIV-related health behaviors.

Community health workers (CHWs) may play a pivotal role in HIV care and prevention in highly mobile communities, by expanding health care access and serving as a trusted source of information and social support. CHWs are members of the community they work in who receive training to promote health-related behaviors and offer linkage to services. With growing evidence establishing the utility of CHWs in low- and middle-income countries (6, 7), the World Health Organization recommends that CHW programs serve as an integral part of health systems (8).

As a potential strategy to increase uptake and adherence to HIV prevention and treatment behaviors in the fishing community of Kasensero, Uganda, Rakai Health Sciences Program (RHSP) began implementing an intervention called Health Scouts in 2015. The intervention employs CHWs, called Health Scouts, to provide one-on-one counseling sessions on HIV prevention and treatment behaviors. Health Scouts use counseling skills informed by motivational interviewing (MI), a communication strategy that emphasizes a nonjudgmental and nonconfrontational approach to mobilize the client’s own motivation to change (9, 10). Target behaviors of the intervention include PrEP use, condom use, HIV testing, engagement in male circumcision, antiretroviral therapy (ART) adherence, and engagement in HIV care services. During each visit, Health Scouts counsel clients with assistance from a smartphone application which offers theory-based, MI-informed prompts for tailored messaging and psychosocial support. The Health Scout intervention design and protocol for the cluster randomized controlled trial has been published elsewhere (4).

The counseling content of the Health Scout intervention was based on the situated Information, Motivation, and Behavioral Skills (sIMB) conceptual framework for engagement in HIV care (11). This framework uses the three core determinants of initiating and sustaining a behavior over time established in the IMB model: information, motivation, and behavioral skills (12, 13). Each dimension is “situated” to reflect the kinds of information, motivation, and skills most relevant to the local socioecological context in which HIV prevention and treatment behaviors are negotiated. *Information* refers to accurate (versus inaccurate) facts and knowledge about a behavior, service or treatment. *Motivation* refers to attitudes and beliefs about the positive and negative consequences of adopting (and of not adopting) the health promoting patterns of behavior at the personal and social levels. *Behavioral skills* refer to the abilities and self-efficacy which guide successful adoption of behavior patterns. Health Scouts were trained to explore each dimension as they relate to HIV prevention and treatment, given a client’s unique perspective and circumstance.

This study is an sIMB-based, qualitative exploration of the content of Health Scout visits from the perspective of clients, to describe what specific information, motivation, and behavioral skills proved relevant for clients when considering behavior change. We situate these elements within the context of self, relationships, community and environment which clients experience in their fishing village. Our study works to characterize clients’ authentic experience with the Health Scout intervention and the applied utility of the sIMB framework. Insights can inform evidence-based content of CHW counseling programs, responding to the call for increased quality in CHW interventions for tailored health promotion and preventive care at the community level (14).

## Methods

### Health Scout Intervention

The site of this study is a large fishing landing site located on Lake Victoria in Rakai District, Uganda. Operating as the primary provider of combination HIV services in this community since 2011, RHSP runs an HIV clinic providing free ART care and management, HIV testing and counseling, and PrEP screening and supply. As part of a randomized trial, RHSP implemented the Health Scout intervention from September 2015 to December 2018. The end-study analysis of primary outcomes found that the Health Scout intervention improved HIV care and ART coverage in intervention clusters compared to control clusters, but did not clearly improve male circumcision coverage or HIV viral suppression (15).

Health Scouts were elected and recruited from the community and underwent trainings on smartphone use, confidentiality, disclosure, HIV-related knowledge, and intervention protocol. Health Scouts also received an initial and refresher training on MI skills, which included roleplay activities to contextualize training to the community’s context. Core competencies of MI counseling were evaluated, and Health Scouts were provided with individualized feedback. Health Scouts were assigned clusters within the community, and attempted to visit all clients within their clusters once every 3 months. During each visit, Health Scouts approached clients at their households and, after receiving oral consent, counseled clients with assistance from the smartphone application prompts (16).

The study population was highly mobile, which presented challenges to implementation. Health Scouts worked to maintain visit continuity despite client mobility by getting in touch by phone or in person ahead of time to schedule visits, giving multiple attempts to locate unfound clients when due for a visit, and designating clients to a new Health Scout if they moved into another intervention cluster.

### Study Procedures

Semi-structured, in-depth interviews were conducted with clients of the Health Scout intervention between September and November 2018. Clients were recruited through random purposeful sampling. We generated a randomized list of participants to interview using a sampling frame of clients in the database who had at least one Health Scout visit documented, stratified by gender, HIV status, Health Scout assignment, and three age categories (18–25, 26–35, 35+) (17). Interview guides focused on overall experience with the intervention, sIMB framework dimensions, interactions with Health Scouts, and context of carrying out health behaviors in the fishing village. Interviews took place in the community and were conducted by trained ethnographers in the Social and Behavioral Sciences department at RHSP who were not involved in program implementation. Informed, written consent was obtained from all participants. Interviews were conducted in Luganda and digitally recorded, then transcribed and translated to English.

Transcripts were iteratively coded in phases to characterize themes. An initial codebook was developed deductively between two researchers, with codes derived from theoretical elements in the sIMB framework and codes to capture themes falling outside of the framework. Over multiple rounds, researchers independently coded the same text segments, or meaningful analytical units (18), and discussed discrepancies across codes. As coding proceeded, additional codes were added inductively to capture contextual factors. We next coded all data across HIV prevention and treatment behaviors (i.e., using PrEP, using condoms, sexual behaviors, HIV testing, and ART adherence) then analyzed themes mapped across sIMB elements and contextual factors. We used QSR NVivo Version 12.3 for data management and coding and Microsoft Excel Version 16.22 for matrix analysis.

## Results

**Figure 1:**
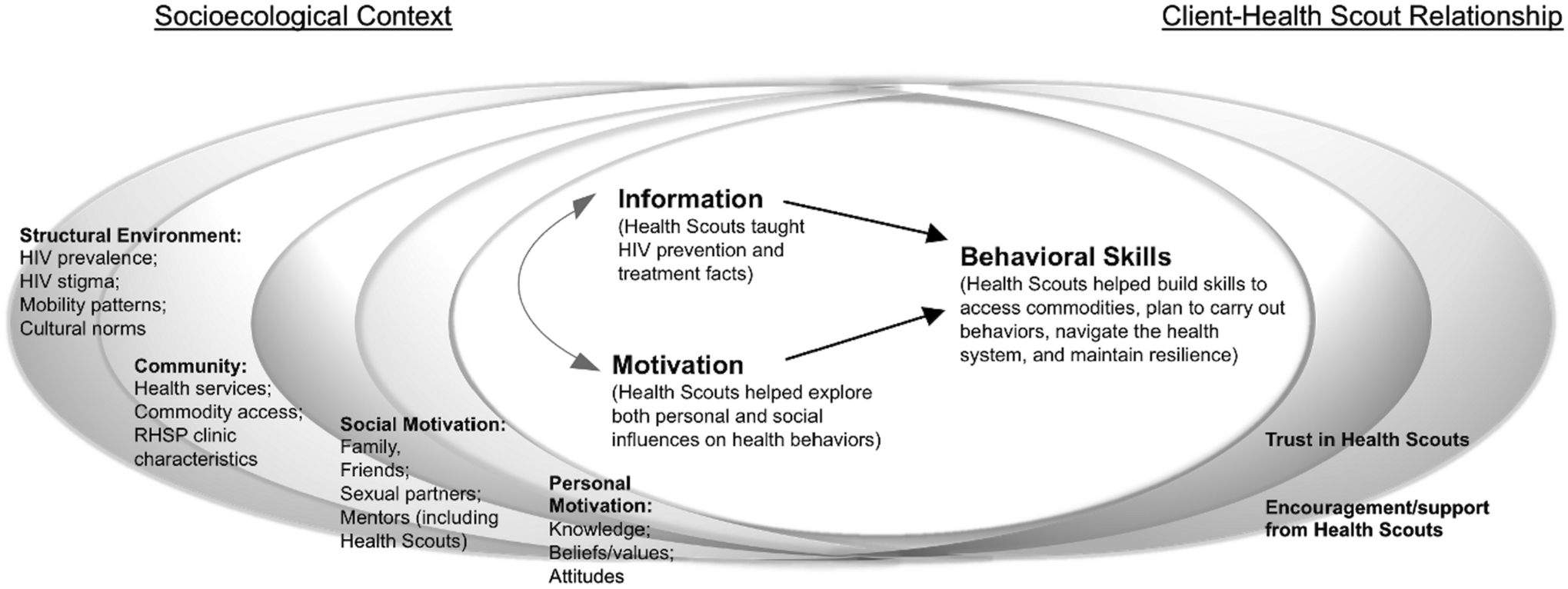
Themes in sIMB dimensions and contextual factors for Health Scout clients

We interviewed 51 clients who had received one or more Health Scout counseling sessions. As shown in Table I, 27 clients self-reported as HIV positive and 24 as HIV negative. We present themes and selected client narratives across information, motivation, and behavioral skills (see Table II for HIV prevention behaviors and Table III for HIV treatment behaviors). Within these tables, we list influential elements reported by clients across the sIMB dimensions and indicate if clients said the element had an activating influence, moving them towards carrying out a health-promoting pattern of behavior (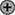) or if the element moved clients away from carrying out a health-promoting pattern of behavior (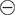). Lastly, we characterize the client-Health Scout relationship and contextual factors as reported by clients, and present how these aspects were inherent in sIMB dimensions affecting behavior change (see Figure 1).

### HIV Prevention

#### PrEP:

Clients reported gaining motivation to acquire and take PrEP from Health Scouts across sIMB dimensions. Many clients first heard about PrEP and where to access it from their Health Scout. Clients were particularly encouraged by the use of PrEP to protect against HIV transmission from condomless sex with their regular HIV positive partner. A major deterrent of initiating PrEP discussed by clients was the daily burden of taking a pill, which clients felt was similar to taking ART. As one client stated, *“I won’t be different from someone who has HIV*.” (Shop keeper, 23 years old)

#### Condoms:

Clients shared that Health Scouts offered accurate information about condoms and helped equip them with skills facilitating consistent condom use. Clients were especially motivated by the protection condoms provided when having sexual encounters outside of their marital relationship. Some Health Scouts supported clients’ access to condoms by delivering them directly to clients or explaining where condom collection boxes were located. Influences which moved clients away from using condoms included the negotiation process with partners and negative perceptions such as decreased sexual pleasure and difficulty in handling condoms. Multiple clients shared how their Health Scout countered these concerns by giving condom demonstrations in their visits and reinforcing the protective benefit of condoms.

#### Sexual Behaviors:

Clients said that Health Scouts helped them understand the connection between decreasing the number of sexual partners and minimizing one’s risk of HIV infection. According to clients, Health Scout counseling particularly influenced personal motivations to change sexual behaviors, with the main personal benefit being the prevention of HIV to live a longer, healthier life. Some clients realized the consequences of their sexual activity on others, predominantly their marital partner, “*If you have a wife, you have to make sure that you protect her so that you stay alive. You shouldn’t have many sexual partners.”* (Fisherman, 40 years old)

Challenges to reducing sexual partners included frequent social gatherings and substance use, which clients described facilitating casual sexual encounters. One client explained, “*Drugs are like alcohol. When you consume it in a group where there are men and women, you can’t fail to hook up with one of them*.” (Shop keeper, 23 years old) Even after Health Scout counseling, interpersonal motivations remained a salient barrier for clients to adopt risk-reducing sexual behaviors.

#### HIV Testing:

Clients spoke about learning new information about HIV testing from Health Scouts, as well as realizing personal benefits and overcoming fears. Clients felt especially reassured after Health Scouts clarified what happens after someone tests HIV positive by providing accurate treatment information. Preventing the consequences of HIV seen in their friends and family members was a key motivator towards testing for some clients.

### HIV Treatment

#### ART Adherence:

Many participants, both who were living with HIV as well as not living with HIV, discussed aspects of HIV treatment. For some clients not living with HIV, familiarization with HIV treatment brought comfort:

I used to see people who had HIV as people who were going to die but I since learned from [my Health Scout] that there is medicine that can get people to live healthily. Now I know that if God brings HIV to me, I can also swallow medicine and live.

(Fisherman, 32 years old)

Many clients living with HIV said Health Scout counseling helped them initiate and maintain proper adherence to ART. Facts clients learned about ART treatment, from the importance of timely, daily dosage to the medicine’s impact on viral load, helped motivate clients towards proper adherence. Health Scouts set realistic expectations for clients and offered ways to reduce side effects such as dizziness and fatigue. This helped resolve the unease and discomfort clients associated with ART, which was severe in some cases:

When I would put the tablets into my mouth, I always thought that I was going to die. The Health Scout helps me stay strong. She visits me so I don’t feel discouraged while taking my [ART] medicine. I don’t feel like my life is over.

(Stall owner, 24 years old)

Clients stated that Health Scouts also encouraged them to consider the advantages of maintaining proper adherence to ART. Clients spoke of gaining personal motivation to take ART from preserving their own health; others gained motivation from observing or learning about others who were living with HIV and taking ART:

[My Health Scout] told me that I wasn’t the first one to fall sick…I said to myself that I am not alone…I must take medicine and follow the health worker’s guidelines so that I stay alive like the others.

(Stall owner, 38 years old)

Clients spoke with their Health Scout about social relationships being an influential component of ART adherence. Spouses who reminded clients to take their ART served as an activating influence towards proper adherence. Yet for clients who had not disclosed their HIV status to their partner, spouses posed a threat to proper adherence since many clients hid taking their ART openly. One woman disclosed that she took ART pills from a latrine every day out of fear her husband would learn she was living with HIV. Other clients feared public appearances at the health clinic:

Most people fear taking their medicine openly. They hide themselves and collect their medicine from other clinics far way…such that other persons cannot easily identify that patient X is taking ART. This is very common here in Kasensero.

(Fisherman, 34 years old)

Multiple clients who feared stigmatization by appearing at the clinic reported that Health Scouts enabled access to ART by retrieving refills and delivering them in their visits. In contrast, other clients voiced strong skills to resist stigma, feeling unashamed and accepted by their community:

I am not afraid of being stigmatized musawo [health worker] because all the people around me know it. When it’s time and we are having a conversation, I tell them that it’s time for me to swallow medicine because I don’t hide it.

(Stall owner, 38 years old)

Health Scouts equipped clients with behavioral skills for ART adherence, facilitating their ability to access ART, navigate systems of care and plan for appropriate adherence. One client living with HIV shared the plan he made with his Health Scout to take his medicine: “*When you go for fishing, you go with enough medicine for the days you are going to spend in the water*.” (Fisherman, 32 years old) For clients, learning behavioral skills helped inspire personal agency and resilience to appropriately treat HIV:

I am confident because it is my life and I cannot play with it. I am not going to exchange it with another person. So, I have to stick on what [the Health Scout] told me, and swallow medicine.

(Farmer, 39 years old)

### Client-Health Scout Relationship

A pivotal component of the intervention demonstrated through client interviews was the support and counseling Health Scouts provided in their visits and the relationships between clients and Health Scouts. Clients reported that the reassurance and supportive problem-solving they received from their Health Scout bolstered their adoption and maintenance of behaviors:

The time [my Health Scout] spends with me makes me happy because in it I am encouraged to keep protecting myself. Also, the things that he reads for me and tells me about makes me stronger.

(Fisherman, 32 years old)

The emotional support and encouragement clients experienced from Health Scout counseling added to social motivations activating HIV prevention and treatment behaviors. Another influential aspect shared by clients was their pre-existing perceptions of Health Scouts. Clients explained that their level of familiarity with and trust of their Health Scout influenced how willing they were to give time for counseling sessions and engage in open discussion.

### Situated Context

Contextual elements described by clients in interviews included community-level factors (i.e., health services and commodities available in the fishing village) and structural factors (i.e., patterns of mobility, cultural norms of substance use, widespread HIV stigma, and perceptions of HIV influenced by the community’s history of high HIV prevalence). These elements are summarized in Figure 1, positioned to show the interrelated nature of the sIMB framework, socioecological context, and client-Health Scout relationship in shaping behavior change.

## Discussion

Using qualitative assessment, we explored client perceptions of a CHW-delivered intervention in Rakai, Uganda to foster HIV prevention and treatment behaviors. Clients shared content from counseling sessions which mapped to sIMB framework dimensions. The conceptual model successfully characterized HIV prevention and treatment information received by clients, their personal and social motivations, and behavioral skills promoted by CHWs, similar to other HIV studies employing IMB dimensions (19, 20). Personal motivations for both clients not living with HIV and clients living with HIV similarly hinged on self-preservation for a long and healthy life; however, social motivations differed between these groups. Clients not living with HIV often discussed with Health Scouts how to navigate the reactions of partners to HIV preventive behaviors, while clients living with HIV explored with Health Scouts their perceptions and experiences of HIV stigma in the context of HIV management. Elements which moved clients away from HIV prevention and treatment behavior change in our study can inform content for future HIV behavioral interventions. These elements included daily pill burdens, serostatus disclosure, fear of testing positive, substance use, and anticipated reactions of partners. An area of high relevance was clients’ level of familiarity with and trust of Health Scouts, which shaped client willingness to engage in open discussion during counseling sessions and entertain ideas of behavior change.

Findings in our study emphasize the defining role of this fishing community’s social and structural environment in shaping client behaviors. This included the high HIV prevalence affecting clients’ knowledge and perceptions about HIV, either through direct observation of others or inherited notions from past generations. Periodic migration is another structural factor with implications for HIV transmission, as moving frequently can disrupt patterns in taking PrEP or ART and interrupt routine health care established at facilities (21). Mobility, particularly in Uganda, has been linked to higher-risk sexual behaviors through expanded sexual networks and engagement in transactional sex (22, 23). These structural realities can be pivotal in navigating HIV behaviors, emphasizing the need for counseling interventions to consider situated context. Components of the Health Scout intervention are well-placed to be re-contextualized to new communities, especially highly mobile and/or HIV-hyperendemic populations; however, programs should ensure content is relevant to the local context in which behaviors are carried out.

Many determinants of behavior change which clients spoke of in our study are similarly reported in other studies conducted in sub-Saharan Africa exploring HIV care retention. In particular, stigma and a fear of disclosure have been identified through systematic reviews as barriers to accessing and remaining in HIV care (24–27). The influential role of client-Health Scout relationships in our study is consistent with previous research reporting how encouraging and supportive relationships with caregivers are associated with HIV care retention. Positive interactions with health workers, involving collaborative decision-making, non-judgmental treatment, and trust, have similarly been shown to enhance sustained engagement in HIV care programs (26, 28). Our study adds to the literature establishing the potential of CHWs to help individuals overcome challenges to healthy HIV-related behaviors, and complements previous studies of community health worker HIV interventions in Rakai, Uganda (29, 30).

There are limitations to our study. First, RHSP did not begin offering PrEP in Kasensero until 2017, two years after the Health Scout intervention began. Clients thus had less exposure to counseling about the use of PrEP compared to other behaviors analyzed in this study. Second, it is possible that clients with more frequent contact with Health Scouts were more likely to be recruited, and those with positive experiences were more likely to accept to be interviewed. Our random purposeful sampling strategy worked to minimize this bias. With our focus in this study on participants who had some exposure to the intervention, we recognize that we cannot speak to attitudes or beliefs among those who chose not to participate in Health Scout counseling visits. Additional qualitative work is underway to better understand these experiences. Lastly, due to the contextualized nature of the sIMB framework, findings from our study may not be fully generalizable to other settings.

## Conclusion

Our study adds to the evidence base establishing CHWs as potentially pivotal mediators to promote the use of PrEP, condoms and ART and facilitate access to healthcare services, while navigating structural realities including HIV stigma, community norms, and frequent mobility. Findings describe the relationship between CHW counseling and behavior change from the perspective of clients which, when tailored to context, can inform future HIV behavioral interventions to optimize client-centered approaches.

## Figures and Tables

**Table I:** Demographic characteristics of participants (n=51)

	Total (%)
Gender	
Women	28 (55%)
Men	23 (45%)
Age	
≤ 25 years	17 (33%)
26 – 35 years	18 (35%)
≥ 36 years	16 (31%)
Marital status	
Married	36 (71%)
Single	13 (25%)
Separated	2 (4%)
Self-reported HIV Status	
Positive	27 (53%)
Negative	24 (47%)
Occupation	
Fishing	18 (35%)
Shop keeping	9 (18%)
Agriculture/Housework	8 (15%)
Bar/restaurant	6 (12%)
Laundry/Tailoring	3 (6%)
Other	3 (6%)
Cow herding	2 (4%)
Sex work	2 (4%)

**Table II:** HIV prevention information, motivation, and behavioral skills clients discussed with Health Scouts

	Information discussed with Health Scouts		Associated client Motivation		Behavioral Skills supporting behavior	Client Example
	Mis-information	Accurate Information	Personal Motivation	Social Motivation		
**Using PrEP** (32 clients coded)	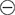 PrEP can cause HIV 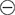 PrEP can cause death 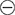 PrEP harms reproductive system	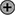 PrEP is a pill taken daily which prevents HIV infection 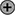 PrEP prevents HIV without use of condom 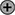 PrEP is available and monitored at health clinic	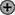 Remaining HIV negative, living longer 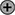 Feeling a sense of safety 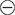 Daily pill burden, fear of side effects	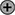 Desire to stay healthy for children or family 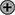 Desire for protection regardless of partner’s sexual behavior or condom use	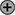 **Access:** Going to health clinic for screening and supply of PrEP 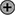 **Resilience:** Confidence in protecting oneself from HIV infection	*“The [PrEP] medicine is working for me because I know the virus has nowhere to pass… [My Health Scout] simply gave me information and I made a choice about how to live my life. So, I decided to swallow PrEP.”* (Fisherman, 25 years old)
**Using condoms** (36 clients coded)		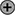 Condoms protect against HIV infection, STIs and pregnancy 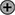 Condoms prevent reinfection from a different strain of HIV 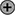 How to correctly use a condom, including proper application and single usage 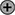 Condoms should be used when having multiple partners and when a partner’s HIV status is unknown	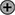 Preventing HIV and STIs, enabling sex with HIV positive partner 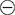 Difficulty putting on a condom 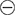 Side effects of decreased sexual pleasure, dryness, pain from friction, itching or abdominal pain	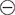 Alcohol use and social gatherings impede condom use 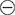 Partner prefers or expects not to use a condom 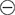 Male partner resists using a condom, potentially forcefully	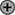 **Access:** Acquiring condoms from collection boxes or Health Scout 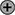 **Planning:** Ensuring you have condoms at time of sexual encounter 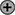 **Resilience:** Autonomy in deciding to use condoms	*“I have learned [from my Health Scout] about things regarding sex. There are times when you may not know someone’s status, in that case you have to use a condom…I started using condoms in every sexual encounter, and refrained from having sex with people who refused to use condoms.”* (Student, 19 years old)
**Sexual behaviors** (37 clients coded)		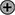 Reducing number of sexual partners decreases HIV risk	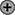 Feeling organized, careful, and healthy 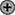 Preventing HIV 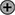 Freedom from fear of acquiring HIV 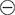 Resisting sexual attraction and sex drive	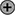 Commitment or emotional connection to a single partner 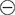 Suspicion of partner infidelity 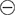 Alcohol use and social gatherings encourage risky sexual behaviors	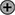 **Planning:** Choosing to reduce number of sexual partners rather than taking PrEP or using condoms, deciding to reduce alcohol intake	*“The challenge we discussed was about getting exposed to HIV reinfection through having sex with sex partners… my attitude and behaviors changed because I stopped having other sex partners. I have spent over five years now without having other sex partners. I only stick to one partner…I adjusted my conduct after talking to my Health Scout.”* (Fisherman, 34 years old)
**HIV Testing** (31 clients coded)		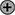 If you test HIV positive, you can live a long and healthy life by adhering to ART 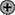 If you test HIV negative, you should maintain HIV prevention behaviors 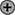 You should get tested for HIV with a new partner before sex	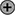 Living a healthy life without worry 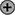 Avoiding health problems observed in others living with HIV 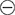 Fear of testing HIV positive	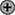 Reassurance and counseling from Health Scout 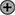 Encouragement from friends or sexual partner 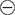 Fear of negative or violent reactions from partner	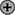 **Systems Navigation:** Knowing where and when to go for HIV testing 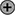 **Planning:** Testing with new partner before sex, planning to get tested for HIV at regular intervals	*“She told me to go to the clinic to find out what my status is although I wasn’t ready to come to the clinic because I was scared. She encouraged me and counselled me and I came to the clinic. I was tested and diagnosed as HIV positive…she comforted me and told me that she is also HIV positive, and she has been on ART for a long time. I found the strength to do the same.”* (Fisherman, 24 years old)

Key: 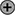 = Influence *towards* HIV prevention behavior change (activating)

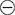
 = Influence *away* from HIV prevention behavior change

**Table III:** HIV treatment information, motivation, and behavioral skills clients discussed with Health Scouts

	Information discussed with Health Scouts		Associated client Motivation		Behavioral Skills supporting behavior	Client Examples
	Mis-information	Accurate Information	Personal Motivation	Social Motivation		
**ART Adherence** (34 clients coded)	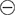 Possible to test HIV negative if taking ART 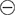 Taking ART will lead to a quicker death 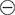 If you are living with HIV and feel healthy, you should not take ART	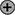 Proper adherence to ART reduces viral load, increases physical strength, elongates one’s life and reduces risk of transferring HIV to sexual partner 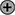 Improper ART adherence leads to health problems and possibly death 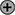 How to minimize side effects of ART	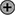 Selfpreservation, feeling energetic and healthy, prolonged work productivity 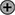 Enabling a healthy appearance and functionality observed in others taking ART 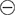 Fear of side effects 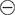 Daily pill burden	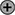 Emotional support and inspiration from Health Scout 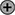 Reminders from friends or spouse 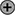 Obligation to take care of children 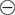 Fear of HIV status disclosure to partner 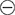 Anticipated stigma and judgement from others when going to the health clinic for appointments and refills	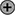 **Access:** Health Scout bringing refills of ART 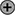 **Systems Navigation:** Knowing where to acquire regular supply of ART, understanding process of clinic transfer 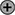 **Planning:** Establishing routine to take ART, anticipating refills, bringing adequate pills when traveling or fishing 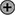 **Resilience:** Feeling confident and firm in lifelong ability to adhere to ART	*“I hope if I take the [ART] medicine very well I will live longer and I can have more children… I cannot fail to stick on it …[the Health Scout] comforts you more than the person who has only given you medicine.”* (Bar owner, 32 years old) *“There was a time I had stopped taking medication and [my Health Scout] told me it’s a very wrong thing to do. So, I started taking my medicine again…The fact that she encourages and comforts me does a lot for my life because I never fall out of line since I have someone following up on me. She will check my file and say that ‘on the 13th you are going for a refill. Are you aware of that?’ I like that…I have to take medicine to survive. [My Health Scout] simply helps me remain on track.”* (Cow herder, 45 years old)

Key: 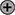 = Influence *towards* HIV treatment behavior change (activating)

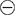
 = Influence *away* from HIV treatment behavior change
